# Case Report of Necrotizing Fasciitis Associated with* Streptococcus pneumoniae*

**DOI:** 10.1155/2016/6872739

**Published:** 2016-03-30

**Authors:** Lei Jiao, Zain Chagla, Reham Mohammedsaeed Kaki, Gabriela Gohla, Marek Smieja

**Affiliations:** ^1^Medical Microbiology Postgraduate Training Program, Pathology and Molecular Medicine, McMaster University, Hamilton, ON, Canada L8S 4L8; ^2^St. Joseph Healthcare and Hamilton Health Sciences, Hamilton, ON, Canada L8N 1Y2; ^3^Department of Medicine, McMaster University, Hamilton, ON, Canada L8S 4L8; ^4^Department of Medicine, King Abdulaziz University, Jeddah, Saudi Arabia; ^5^Hamilton Health Sciences, Hamilton, ON, Canada; ^6^Department of Pathology and Molecular Medicine, McMaster University, Hamilton, ON, Canada L8S 4L8

## Abstract

Necrotizing fasciitis, caused by* Streptococcus pneumoniae*, is an extremely rare and life-threatening bacterial soft tissue infection. We report a case of early necrotizing fasciitis associated with* Streptococcus pneumoniae* infection in a 26-year-old man who was immunocompromised with mixed connective tissue disease. The patient presented with acute, painful, erythematous, and edematous skin lesions of his right lower back, which rapidly progressed to the right knee. The patient underwent surgical exploration, and a diagnosis of necrotizing fasciitis was confirmed by pathological evidence of necrosis of the fascia and neutrophil infiltration in tissue biopsies. Cultures of fascial tissue biopsies and blood samples were positive for* Streptococcus pneumoniae*. To our knowledge, this is the first report of necrotizing fasciitis resulting from* Streptococcus pneumoniae* diagnosed at early phase; the patient recovered well without surgical debridement.

## 1. Introduction

Necrotizing fasciitis is an uncommon soft tissue infection involving the skin, subcutaneous tissue, and fascia. The mortality rate ranges from 25% to 35% despite broad-spectrum antibiotic therapy and surgical debridement [1]. Typically, this is caused by a variety of pathogens such as *β*-hemolytic group A streptococcus (GAS),* Staphylococcus aureus*,* Klebsiella pneumonia*,* Clostridium* species,* Fusobacterium* species, and* Prevotella* species [2]. The infection may start along the fascial plane and cause symptoms including erythematous, painful, and edematous skin lesions which often rapidly deteriorate to hemorrhagic blisters, anesthesia, and gangrenous necrosis over several days. This process is usually accompanied by systemic toxic manifestations such as septic shock, decreased level of consciousness, and multiorgan failure [3].

*Streptococcus pneumoniae* is well recognized as a common cause of pneumonia, bacteremia, and meningitis. However, this is an extremely rare etiologic agent for necrotizing fasciitis. Only 20 cases were previously reported among patients with underlying diagnosis such as systemic lupus erythematosus (SLE), human immunodeficiency virus (HIV) infection, and diabetes mellitus [4–6]. Here, we report a case of necrotizing fasciitis resulting from* Streptococcus pneumoniae* evidenced by clinical presentation and pathological findings.

## 2. Case Report

A 26-year-old man was admitted with a 6-hour history of rapidly developing erythematous, severely painful, mildly to moderately edematous skin rash. The rash started from his right lower back and spread within hours to include the right hip, abdomen, and groin, and down to the proximal third of the anterior lateral aspect of right thigh. At the time of initial infectious diseases consultation, the skin lesion had further spread from the upper third of his right thigh to the upper edge of his right knee over the course of 40 minutes. The patient complained of extreme fatigue; however, he denied any fever, nausea, vomiting, coughing, shortness of breath, dysuria, or sick contacts. He also denied any history of allergy, tobacco use, alcohol consumption, or intravenous drug use. His medical history revealed a history of a mixed connective tissue disease (limited systemic sclerosis overlapping with polymyositis and SLE which had been diagnosed one month previously). He was currently being treated with intravenous immune globulin (IVIG) every three months and prednisone 40 mg daily. He had received a pulse infusion of 1 gram of cyclophosphamide 18 days prior to the current presentation.

His initial vital signs were stable, with temperature of 36.2°C, blood pressure of 123/82 mmHg, heart rate of 114/min, respiratory rate of 22/min, and oxygen saturation of 92% on room air. The patient was alert and oriented. Further physical examination revealed marked erythema over his right lower back, right hip, abdomen, and groin area, as well as the anterior lateral aspect of right thigh. The overlying skin was warm, mildly edematous, and extremely tender when palpated. No sign of crepitus was identified. The patient was neurovascularly intact in both lower limbs. Laboratory studies included a white blood cell count of 6.0 × 10^9^/L, absolute neutrophil of 5.8 × 10^9^/L with left shift noted, hemoglobin of 94 gm/L, platelets of 174 × 10^9^/L, sodium of 130 mmol/L, potassium of 4.1 mmol/L, creatinine of 58 *μ*mol/L, lactate of 2.9 mmol/L, creatine kinase of 661 U/L, and C-reactive protein of 99 mg/L. His hepatic profile revealed elevated alanine aminotransferase of 83 U/L and alkaline phosphatase of 321 U/L. Coagulation study showed normal prothrombin time and an elevated partial thromboplastin time of 47 seconds (22–35 seconds).

**Figure 1:**
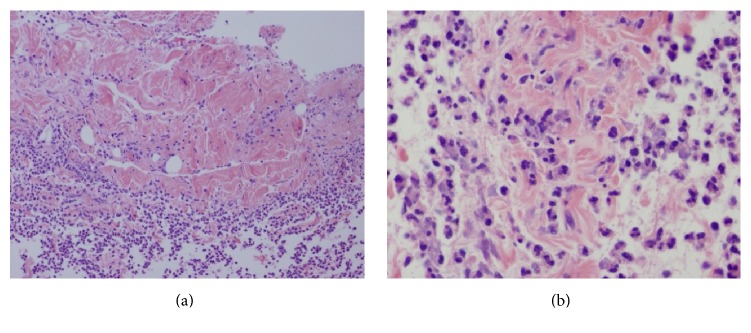
Pathological findings of fascia necrosis and neutrophil infiltration in the fascia tissue. (a) 100x. (b) 400x.

The patient was empirically started on piperacillin-tazobactam 4.5 gm IV every 6 hours and vancomycin 500 mg IV every 12 hours which cover broadly for Gram negative and Gram positive organisms including methicillin-resistant* Staphylococcus aureus* (MRSA), along with clindamycin 600 mg IV every 8 hours. Clindamycin was given to inhibit the toxin production from possible invasive* Streptococcus pyogenes* based on available human and animal studies [7–9]. One dose of IVIG was given since he was due for his routine dose for his rheumatological disorders, and given that IVIG may improve outcomes of necrotizing fasciitis from GAS infection [8]. Tissue biopsy of fascia from the anterior and lateral side of right thigh was performed by the orthopedic surgery team at the bedside under local anesthesia and was sent for urgent frozen section pathology and microbiology. Both tissue and blood sample cultures grew* Streptococcus pneumoniae* sensitive to penicillin. Pathological findings with heavy neutrophilic infiltration and abundant necrosis within fat and fascia were consistent with necrotizing fasciitis. Contrast computerized tomography of the abdomen and pelvis was performed after tissue biopsy suggesting postbiopsy changes without obvious infection involving fascia or muscles. Based on the patient's rapidly developing clinical symptoms and evidence of necrotizing fasciitis on tissue biopsy, he was taken to the operating room urgently. During surgery, he was found to have extensive edema of his subcutaneous tissue, but with no obvious fascial or muscle necrosis. Therefore, no debridement was performed. Fascial tissue from the anterior and lateral side of right thigh was randomly sampled intraoperatively and clear evidence of necrotizing fasciitis with significant neutrophilic infiltration and necrosis in fat and fascia was confirmed by pathological results, as shown in Figure 1. Clindamycin and vancomycin were discontinued on the second day after the microbiology culture results became available. Piperacillin-tazobactam was switched to ceftriaxone which was continued to complete the course of 6 weeks of treatment. The patient required 8 days of postoperative ICU stay which was complicated by methicillin-sensitive* Staphylococcus aureus* (MSSA) pneumonia, coagulopathy with factor XI deficiency, and a flare-up of his rheumatologic condition. He recovered well with subsequent physiotherapy on the medicine ward and later in the rehabilitation unit.

## 3. Discussion

Necrotizing fasciitis due to* Streptococcus pneumoniae* is extremely rare, with only 20 cases reported in the English literature by April 2014, of which 11 patients died despite extensive surgical debridement and broad-spectrum antibiotic treatment [5]. Previously reported predisposing factors included rheumatoid arthritis, SLE, type 2 diabetes mellitus, renal transplantation, injection drug use, immunosuppressive therapy, and intramuscular injection with nonsteroidal anti-inflammatory drugs [1, 5]. Here, we report a case of early diagnosed necrotizing fasciitis due to* Streptococcus pneumoniae* in a 26-year-old gentleman who was immune-compromised with prednisone/cyclophosphamide treatment for his severe connective tissue disease. To our knowledge, this is the first report of* S. pneumoniae*-associated necrotizing fasciitis diagnosed at such an early stage of disease that fascial necrosis was found by pathological examination but not by visualization under surgical exploration, which recovered without surgical debridement.

Early recognition and prompt intervention are critical to reduce morbidity and mortality rates. Physicians need to keep a high index of suspicion for necrotizing fasciitis since any delay in diagnosis results in greater soft tissue loss and mortality. The clues indicative of necrotizing fasciitis in the very early stages include pain out of proportion to physical findings, the rapid progression of the disease, systemic toxic symptoms, abnormal findings from imaging tests (mainly computed tomography scan), and the bedside frozen section tissue biopsy [1, 10, 11].

However, it is challenging to distinguish necrotizing fasciitis from other soft tissue infections due to nonspecific skin findings in the very early stage. In a study of 22 patients with necrotizing fasciitis, 59% were found to only have erythematous and tender skin lesions on day 0, whereas signs more suggestive of necrotizing fasciitis such as crepitus and frank necrosis occurred in fewer than 5% of patients on day 0, and in 68% by day 4 [12]. Pain out of proportion to the physical findings was considered to be the most consistent finding in the early stage, which was found in 98% of patients with necrotizing fasciitis on admission [5]. The clinical manifestations of our patient agreed with the first stage of necrotizing fasciitis proposed in the aforementioned study, characterized by relatively stable vital signs, rapidly developing intense erythema, and disproportionate pain [12].

Systemic toxicity such as fever, hemodynamic instability, and multiorgan failure is not a reliable diagnostic clue in the early stage of disease development even though these signs are often stressed in describing necrotizing fasciitis [1]. As reported in the retrospective study of Wong et al., 47.2% of necrotizing fasciitis patients, especially the immunocompromised ones, lack signs of fever and only 4.5% had multiorgan failure on admission [11]. Our findings of a normal temperature and white count in the first 8 hours of disease onset agreed with this report. Of note, our patient did show some level of systemic toxicity, including tachycardia, elevated levels of lactate, CK, CRP, and liver enzymes, and decreased sodium and hemoglobin.

Imaging tests, such as CT or magnetic resonance imaging studies (MRI), provide important evidence supporting clinical diagnosis and, however, were found to have low positive and negative predictive values in the setting of suspected NF [11]. CT is by far the most commonly used diagnostic method. Soft tissue gas is characteristic; however, it was only present in 50% of NF cases in one study [13]. MRI was found to have higher sensitivity than specificity in the diagnosis of NF [14, 15]. In the study of Loh et al. [15], the hyperintense T2 signal from deep fasciae planes and muscle was present in 22 cases; however, only 1 of these was confirmed to be NF. The remaining cases were found to have either abscesses or other noninfectious conditions, 6 of which underwent unnecessary surgeries based on MRI results. Imaging tests may not reliably exclude early disease or differentiate from other conditions; therefore, surgical exploration should not be delayed if clinically indicated.

Bedside biopsy often provides physicians with valuable clues in making an early diagnosis and facilitating timely treatment. In a retrospective review by J. Majeski and E. Majeski [10], 43 patients with suspected necrotizing fasciitis received bedside biopsy under local anesthesia and immediate pathological examinations. Twelve of them were diagnosed as necrotizing fasciitis with a survival rate of 100% which was dramatically higher than the reported rate of 40–80%. The benefit of bedside biopsy was also supported by the work of Loh et al. [15], which found an improved survival rate in patients who were identified as necrotizing fasciitis with the utility of frozen section biopsy compared with those diagnosed with clinical symptoms. This highlights the key role of the bedside incisional biopsy in the early recognition and appropriate treatment, especially with unequivocal evidence from visual examination of the tissue as in our case. Of note, possible false negative results can result from insufficient sampling.

Through the review of 20 reported cases of* S. pneumoniae*-associated necrotizing fasciitis in the literature, symptoms and progression were similar compared with those caused by other bacteria such as* Streptococcus pyogenes* or* Staphylococcus aureus*. Extensive surgical debridement and susceptibility-guided antibiotic treatment as well as aggressive supportive care are usually necessary to treat this devastating disease. High mortality rate of 80% was found in patients without surgical interventions [16]. By contrast, our patient recovered well on antibiotics and supportive therapy without extensive debridement. This could be due to the early and aggressive antibiotic treatment, which stresses the importance of early diagnosis and treatment of necrotizing fasciitis.
